# Supplementary material for: Msh Pilus Mutations Increase the Ability of a Free-Living Bacterium to Colonize a Piscine Host
Source: Genes (Basel). 2021 Jan 20;12(2):127. doi: 10.3390/genes12020127 (PMC7909257; doi:10.3390/genes12020127)
Supplement: Supplementary file 1 [file genes-12-00127-s001.zip › 20210119_mshL_SuppFigures_JFL_Rev2.docx]

***Supplements:***
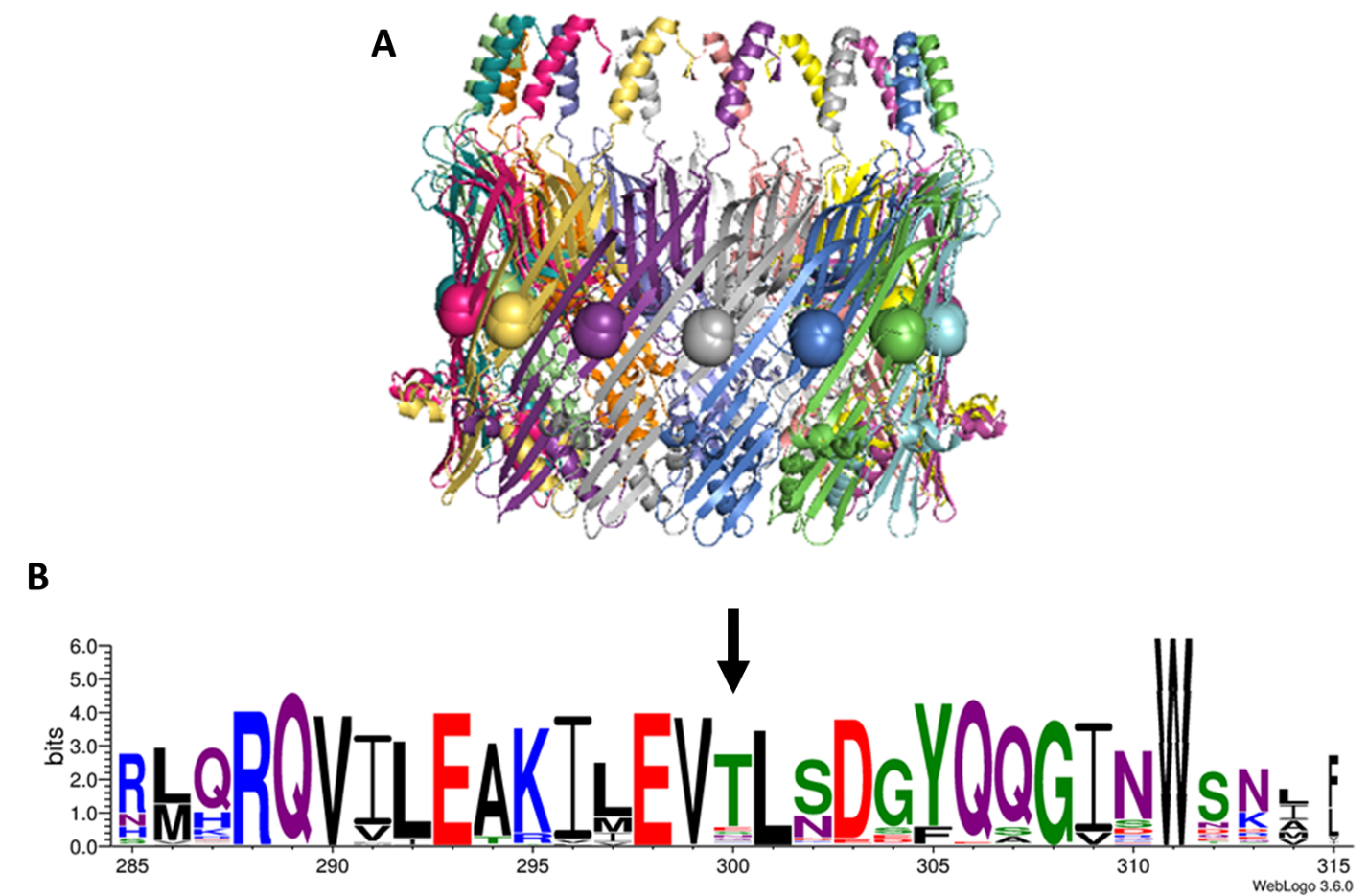


**Figure S1: Location and representation of MshL amino acid 300 in orthologous proteins.** A) Shown is the substructure of the pentadecameric pore formed by ExeD from residues 209-479. The full structure can be found using accession 6I1X in the Protein Data Bank (https://www.rcsb.org/structure/6I1X). Each ExeD chain that makes up this pentadecameric pore is represented by a different color. The enlarged residues, depicted in beta-sheets within each ExeD chain, indicates the position of an alanine at residue 243 which corresponded to the MshL threonine at residue 300 when the ExeD and MshL proteins were aligned. B) WebLogo 3.6.0 (Crooks et al., 2004) was used to depict the amino acid representation among 1247 orthologs around the 300^th^ residue position of MR-1’s MshL protein. An arrow points to the 300^th^ position (site of MshL-T300P mutation). The height of the stacks at each position indicates the level of conservation which can be influenced by a difference in amino acids represented, or the absence of an amino acid in certain orthologs. The height of symbols featured within each stack indicates the relative frequency of an amino acid at that position. Stack widths indicate the proportion of orthologs without gaps for each position (stacks with values for all 1247 orthologs will have the widest widths). Symbol colors: green: polar, purple: neutral, blue: basic, red: acidic, black: hydrophobic.

| **Amino Acid** | **Orthologous representation corresponding to MR-1 MshL-300** |
| --- | --- |
| Arginine | 1.7% |
| Histidine | 0.1% |
| Lysine | 0.3% |
| Aspartic acid | 0.2% |
| Glutamic acid | 4.8% |
| Serine | 3.7% |
| Threonine | 79.3% |
| Asparagine | 3.4% |
| Glutamine | 3.2% |
| Cysteine | 0.0% |
| Glycine | 0.5% |
| Proline | 0.0% |
| Alanine | 1.7% |
| Isoleucine | 0.5% |
| Leucine | 0.0% |
| Methionine | 0.0% |
| Phenylalanine | 0.0% |
| Tryptophan | 0.0% |
| Tyrosine | 0.0% |
| Valine | 0.6% |

**Table S1: MR-1 MshL-300 representation.** The amino acid representation of 1247 MshL orthologues at MR-1 MshL-300 after alignment to MR-1 MshL. Positions 285-315 of MR-1 MshL were used to generate a list of orthologs that shared 70-90 percent identity.


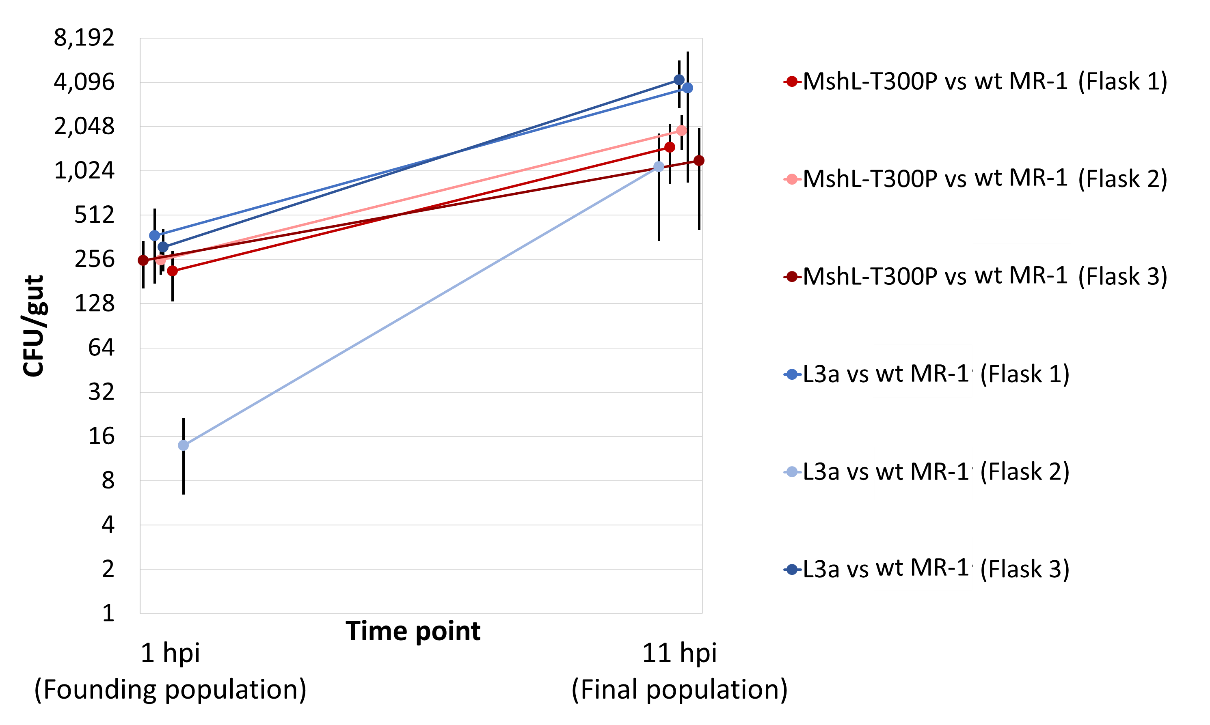


**Figure S2: *In vivo* populations dynamics.** Shown is the mean number of colony-forming units determined for larval guts dissected at 1 hour post inoculation (hpi; founding population) and 11 hpi (final population). Each point represents the mean value of 10 dissected guts for a single flask. Y-axis is ${log}_{2}$-based. Error bars represent the 95 % confidence intervals.

**Table S2: Evolved mutations.** Mutations for each of six replicate evolved populations after serial passage are shown. A-D indicate that four isolates were collected from each replicate population and sequenced. Red and green shading of isolates indicates that each isolate contained a neutral dTomato or green fluorescent protein tag; used to facilitate detection of selective sweeps [7]. X indicates the presence of an evolved mutation. Yellow shading indicates a mutation in the *msh* operon.


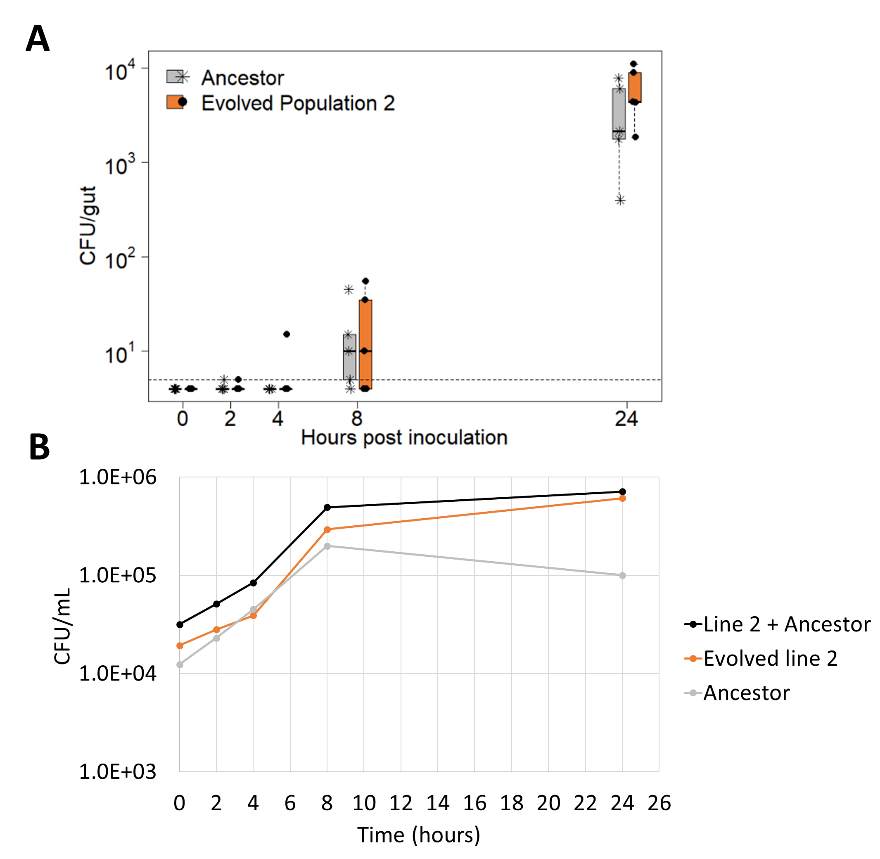


**Figure S3: Colonization and external population dynamics over time.** A) Colonization density over time. A larval GF larval flask was inoculated with competition mixture containing evolved population 2 (orange boxes) and MR-1wt (gray boxes). Five larval guts were then dissected and plated at each indicated time point. Each point shown represents the colonization density of a single gut. The dotted line indicates the limit of detection. B) Same experiment as in A) except that cell densities (CFU/mL) were measured in the EM outside the larvae over the same period.


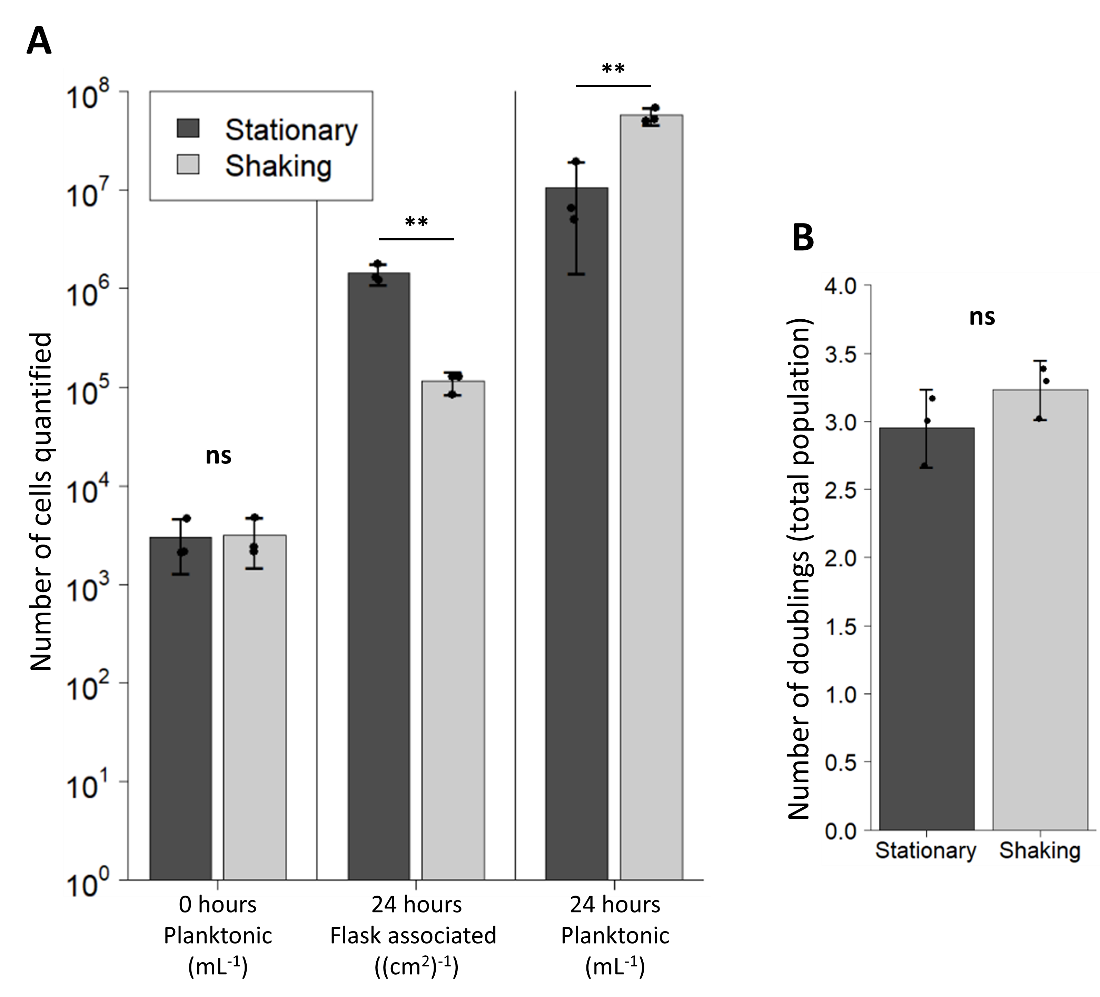


**Figure S4: Planktonic and near-flask population dynamics.** A) Number of cells quantified near the flask surface (flask associated) and in the water column (planktonic) under static and shaking conditions in larvae-conditioned medium (LCM). Flask associated cells were quantified from images of the flask surface. Cells in focus were counted using an automated cell tracking algorithm. Planktonic cells were quantified from colony-forming units (CFUs) resulting from water column samples that were plated. B) Number of total population (planktonic + flask associated) doublings in LCM under shaking and static conditions. For A) and B), populations are the sum of the L3a isolate and ancestral populations competing against each other.
